# The synergistic effects of clopidogrel with montelukast may be beneficial for asthma treatment

**DOI:** 10.1111/jcmm.14239

**Published:** 2019-03-23

**Authors:** Hoang Kim Tu Trinh, Thuy Van Thao Nguyen, Youngwoo Choi, Hae‐Sim Park, Yoo Seob Shin

**Affiliations:** ^1^ Department of Allergy and Clinical Immunology Ajou University School of Medicine Suwon South Korea; ^2^ Department of Pediatrics University of Medicine and Pharmacy at Ho Chi Minh City Ho Chi Minh City Vietnam; ^3^ Department of Biomedical Science Ajou University School of Medicine Suwon South Korea

**Keywords:** asthma, clopidogrel, cysteinyl leukotriene receptors, eosinophil, montelukast, P2Y12R, rodent

## Abstract

Platelets modulate asthma pathogenesis by forming the platelet‐eosinophil aggregation (PEA), which facilitates the activation of eosinophils. Platelets exhibit the purinergic receptor (P2Y12R), which responds to cysteinyl leukotriene E4 (LTE_4_). We have suggested that the combination of an antiplatelet drug (clopidogrel, [Clo]) and montelukast (Mon) would synergistically suppress asthma. BALB/c mice were intraperitoneally sensitized with ovalbumin (OVA) on days 0 and 14 and subsequently challenged on days 28‐30 and 42‐44. Mice were administered with Clo (10 mg/kg), Mon (10 mg/kg) or both drugs (Clo/Mon) orally 30 minutes before the OVA (1%) challenge on days 42‐44. Mice were assayed for airway hyper‐responsiveness (AHR) to methacholine and airway inflammation. Clopidogrel and montelukast attenuated the increased AHR; the combined treatment was more effective than a single treatment for total and eosinophil counts (all *P < *0.05). Levels of interleukin (IL)‐4, IL‐5, IL‐13, platelet factor 4, eosinophil peroxidase and LTE_4_ increased in the bronchoalveolar lavage fluid of asthmatic mice, but these levels decreased in mice treated with Clo/Mon (all *P < *0.05). Goblet cell hyperplasia decreased in response to Clo/Mon. Mouse platelets and eosinophils were isolated and co‐cultured for an in vitro assay with 10 µmol/L adenosine diphosphate (ADP), LTE_4_ (200 nmol/L), Mon (1 µmol/L), Clo (1 µmol/L) and Clo/Mon (1 µmol/L). Flow cytometry revealed that the increased formation of the PEA (%) was fully mediated by ADP and partly mediated by LTE_4_. Clo/Mon reduced ADP‐induced PEA formation and P‐selectin expression (*P < *0.05). In conclusion, Clo/Mon synergistically relieved asthma by inhibiting ADP‐mediated PEA formation.

## 1 INTRODUCTION

Platelets are non‐nucleated blood cells derived from bone marrow megakaryocytes and are indispensable for haemostasis and thrombosis.^1^, ^2^ During the last few decades, platelets have gained increasing attention as effector cells and a bridge between the adaptive and innate immune responses during allergic inflammation.^3^, ^4^ Recent data show that platelets contribute to the allergic response as an immunological barrier through multiple mechanisms. Platelets express various adhesion molecules and surface receptors on their membranes, such as P‐selectin (CD62P), which recruits neutrophils, monocytes and lymphocytes via its ligand, P‐selectin glycoprotein ligand‐1 (PSGL‐1).^3^, ^5^ Platelets store intracellular immune‐associated molecules, such as α‐granules, which are dense granules containing inflammatory cytokines.^1^ Platelets destroy pathogens and directly induce bronchoconstriction through the antimicrobial proteins in the α‐granules, such as platelet factor 4 (PF4) and the pathogenic cytokines.^1^, ^6^ The surface molecules of platelets bind to the surfaces of neutrophils, eosinophil and lymphocytes via ligands, thereby forming platelet‐adherent leucocytes that subsequently amplify cysteinyl leukotriene (LT) production.^7^ Platelets store interleukin (IL)‐33, which is crucial for eosinophilic inflammation in the asthma mouse model.^8^ The hypertrophic factors and extracellular matrix enzymes released from platelets induce smooth muscle hyperplasia and collagen deposition as features of airway remodelling.^2^

Increasing evidence has shown the role of platelets in asthma. Platelets are more activated in patients with bronchial asthma than in healthy controls,^9^, ^10^ although some patients present a mild haemostatic defect.^11^, ^12^ Activated platelets release intracellular mediators and produce microparticles, which increase in patients with asthma.^13^, ^14^ A randomized, double‐blind, placebo‐controlled crossover study demonstrated that prasugrel slightly improves airway responsiveness to mannitol.^15^

The association between platelets and eosinophils has been partially elucidated. Activated platelets adhere to eosinophils through P‐selectin/PSGL‐1, CD40/CD40L and glycoprotein IIb/IIIa/MAC‐1,^8^ forming platelet‐eosinophil aggregations (PEA). Additionally, platelet‐leucocyte aggregations increase after allergen challenge, and the percentages of eosinophil‐bound P‐selectin and eosinophil‐bound αIIb are correlated with the lung function of patients with asthma.^16^ Platelets and eosinophils share similar surface receptors, including the cysteinyl LT receptor type 1 (CysLTR1) and the P2Y12 purinergic receptor (P2Y12R). Adenosine diphosphate (ADP), a specific platelet activator, triggers degranulation of eosinophils through P2Y12R.^17^ Our recent study suggested an inhibitory effect of clopidogrel (Clo), an antiplatelet drug that targets the P2Y12R, during eosinophilic inflammation and degranulation.^18^ Furthermore, LTE_4_, a P2Y12R and CysLTR1 agonist, induces eosinophilic inflammation following a platelet‐dependent mechanism.^19^

We have suggested that LTE_4_ amplifies the activation of platelets and eosinophils, thereby initiating the PEA and subsequent airway inflammation. Furthermore, co‐administering antagonists of CysLTR1 (montelukast, [Mon]) and P2Y12R (Clo) may synergistically enhance the treatment of allergic asthma. Therefore, we established an eosinophilic asthma mouse model and evaluated the synergistic effect of these two drugs. We also investigated the mechanism of the synergistic effect.

## 2 MATERIALS & METHODS

### 2.1 Species and ethics statement

Female BALB/c mice (6 weeks old; weight, 20 ± 2 g) were obtained from the Jackson Laboratory (Bar Harbor, ME). The animals were housed under specific pathogen‐free conditions and were maintained on a 12‐hour light/dark cycle with food and water provided ad libitum. All animal experiments conducted in this study were approved by the Institutional Animal Care and Use Committee of Ajou University (IACUC 2015‐11‐09).

### 2.2 Experimental procedure for secondary challenge, eosinophilic asthma mouse model and drug administration

BALB/c mice were intraperitoneally sensitized with a 10 µg/1 mg ovalbumin (OVA)/aluminium hydroxide solution on days 0 and 14. The mice underwent the primary allergen challenge with 0.2% OVA for 20 minutes on days 28, 29 and 30 using an ultrasonic nebulizer (NE‐SM1; Ktmed Inc, Seoul, South Korea). The mice received a secondary challenge of 1% OVA aerosol on days 44‐46. The mice were given either Clo (10 mg/kg) orally, Mon (10 mg/kg) orally or dexamethasone (Dex) (1 mg/kg) intraperitoneally 30 minutes prior to the OVA challenge. The mice were assayed for further experiments 48 hours after the last challenge. The negative control group mice received sterile 1× phosphate‐buffered saline (PBS) containing dimethyl sulfoxide as the solvent control.

### 2.3 Airway resistance measurement and sample collection

The FlexiVent system (Scireq, Montreal, QC, Canada) was applied to measure airway resistance. On the indicated day, the mice were anaesthetized with pentobarbital sodium and intubated with a cannula. After connecting the mice to a computer‐controlled small‐animal ventilator, the mice were ventilated with a tidal volume of 10 mL/kg at a frequency of 150 breaths/min. The baseline airway resistance (R_L_) of each mouse was recorded. Subsequently, a dilution series of acetyl‐β‐methylcholine chloride (MCh) from 1.56 to 25 mg/mL was gradually introduced to the mice, and the R_L _values at each concentration were recorded.

### 2.4 Harvest of bronchoalveolar lavage fluid and differential cell count analysis

After measuring R_L_, bronchoalveolar lavage (BAL) fluid was harvested with a wash of 1 mL of PBS plus 1% bovine serum albumin (Sigma Aldrich, St. Louis, MO). The BAL fluid was centrifuged at 500 g for 5 minutes at 4°C. The supernatant was collected and stored at −70°C until further analysis. The cells were re‐suspended in 1× PBS.

### 2.5 Measurement of cytokine levels

The BAL fluid levels of IL‐4, IL‐5 and IL‐13 (eBioscience, San Diego, CA); PF4 (Abcam, Cambridge, UK); and eosinophil peroxidase (EPX) (MyBiosource, Inc, San Diego, CA) were measured by ELISA according to the manufacturer's instructions. Serum and BAL fluid levels of LTE_4 _were measured using the Leukotriene E4 ELISA Kit (Cayman Chemical, Ann Arbor, MI).

### 2.6 Identification of PEA in BAL fluid and whole blood of mice

Flow cytometry of PEA in whole blood was performed as described previously.^20^, ^21^ Mouse whole blood was harvested by cardiac puncture into tubes containing 3.8% sodium citrate to prevent coagulation. The whole blood was incubated with phycoerythrin (PE)‐conjugated anti‐mouse Siglec‐F and fluorescein isothiocyanate (FITC)‐conjugated anti‐mouse CD61 for 30 minutes at room temperature (RT) in the dark. Next, red blood cells (RBCs) were lysed with the RBC Lysis/Fixation solution (Biolegend, San Diego, CA) for 10 minutes and washed once with 1× PBS. The cells were analysed immediately by flow cytometry with the BD FACSCanto II (BD Bioscience, San Diego, CA), as described previously.^22^ A leucocyte gate was set based on size and granularity. Within the leucocyte gating, eosinophils were labelled by PE‐conjugated antimouse Siglec‐F. PEA was identified as CD41^+^ eosinophils (Siglec‐F^+^/CD41^+^), and at least 1000 events were recorded for each sample. A pooled sample from three mice was used to obtain a sufficient number of eosinophils for the analysis.

In some experiments, PEA was visualized using double immunofluorescence staining. BAL cells were labelled with anti‐P‐selectin and anti‐EPX antibodies as markers of activated platelet and eosinophils, respectively, and counterstained with mounting medium for the fluorescent‐containing 4′,6‐diamidino‐2‐phenylindole (Vector Laboratories, Burlingame, CA). Fluorescent signals were visualized with Zeiss Zen Microscope software.

### 2.7 Isolation of mouse eosinophil and platelets

The mouse eosinophils were isolated using a previous protocol with some modifications.^23^ Single‐cell suspensions were incubated with peridinin chlorophyll protein complex (PerCP) conjugated with anti‐Ly6G and allophycocyanin (APC)‐conjugated anti‐CD11c antibodies for 30 minutes. Mouse eosinophils were sorted using a BD FACSAria III fluorescence‐activated cell sorter (BD Bioscience). The leucocyte population was identified as the FCS^+^/SSC^+^ cells, and the Ly6G^‐^/CD11c^‐^ eosinophils were sorted out and suspended in RPMI‐1640 medium supplemented with 10% foetal bovine serum, 100 U/mL penicillin G sodium, and 100 µg/mL streptomycin sulphate (all from Gibco, Grand Island, NY) until further experiments were conducted (Figure S1).

In parallel, mouse platelets were isolated as described previously.^20^. Whole blood was centrifuged at 1000 g for 15 minutes at RT. The platelet‐rich plasma (PRP) was collected and centrifuged at 400 *g* for 10 minutes at RT to isolate the platelets. The cells were primed with CaCl_2_ (Sigma Aldrich) (final concentration = 5 mmol/L) for 10 minutes prior to the experiment.

### 2.8 In vitro* PEA assay*

Based on platelets and eosinophils having the same ligands,^24^ mouse eosinophils and platelets were co‐cultured’ stimulated with ADP (Sigma Aldrich), LTE_4_, and LTC_4_ (both from Cayman Chemical) and treated with Mon (1 µmol/L) and Clo (1 µmol/L) for 3 hours. The cells were incubated with PE‐anti mouse Siglec‐F, FITC‐antimouse‐ CD41, and PE/Cy7‐antimouse‐CD62P for 20 minutes. Fluorescent signals were analysed immediately by flow cytometry, as described in the previous section, and at least 1000 events were recorded.

### 2.9 Antibodies and reagents

The antibodies used against the mouse target proteins were anti‐P‐selectin antibody (sc‐8419; Santa Cruz Biotechnology, Dallas, TX), anti‐EPX antibody (sc‐19147; Santa Cruz Biotechnology), Alexa Fluor 488‐conjugated goat anti‐rabbit IgG and Alexa Fluor 594‐conjugated rabbit anti‐goat IgG (ThermoFisher Scientific, Waltham, MA). PerCP‐conjugated anti‐Ly6G (127654; Biolegend), APC‐conjugated CD11c (117309; Biolegend), PE‐conjugated anti‐Siglec‐F (552126, BD Bioscience), FITC‐conjugated anti‐CD41 (133904; Biolegend) and PE/Cy7‐conjugated anti‐CD62P (148310; Biolegend) antibodies were used for flow cytometry and cell sorting. All drugs used for treatment were obtained from Sigma‐Aldrich, except otherwise indicated.

### 2.10 Statistical analysis

Data are presented as means ± standard error. Differences between multiple treatment groups were analysed by one‐way analysis of variance and Tukey's post hoc test, except otherwise indicated. The differences between the stimulated and controls cells in the in vitro assay were analysed using Wilcoxon signed‐rank test. Correlations between cell count with levels of metabolite and chemokines were analysed by Spearman's rank correlation coefficient. The statistical analysis was performed with spss version 23.0 software (spss Inc, Chicago, IL), and a *P* value <0.05 was considered significant.

## 3 RESULTS

### 3.1 Clo/Mon suppresses AHR and the inflammatory cell count in BAL fluid

**Figure 1:**
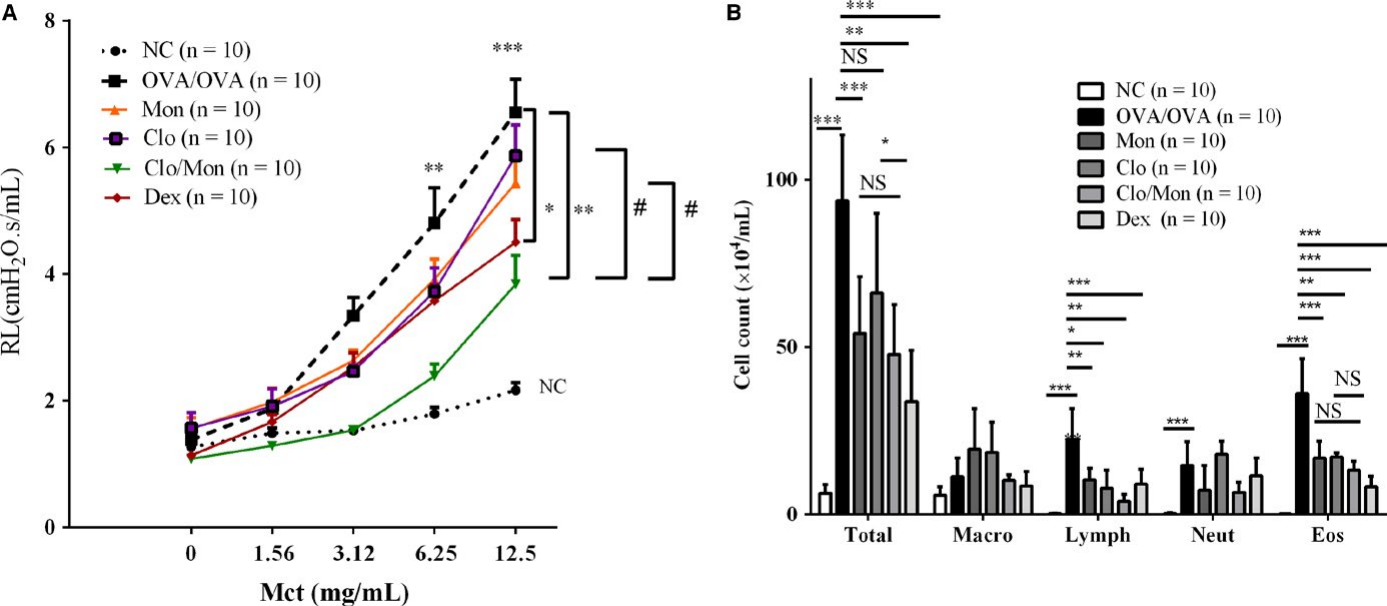
Additional effects of clopidogrel and montelukast (Clo/Mon) in reducing airway hyper‐responsiveness and inflammation. A, Airway hyper‐responsiveness was recorded by the FlexiVent 48 h after the last challenge. B, Total and differential cell counts were calculated using a haemocytometer. *P *values were analysed by one‐way ANOVA with Tukey's post‐hoc test. *, **, and *** indicate *P < *0.05, 0.01 and 0.001, respectively, in the comparisons between the indicated groups; ^#^ *P < *0.05 in comparisons involving the drug‐treated groups; NS, not significant

Mice that received clopidogrel and montelukast (Clo/Mon) showed a further decrease in airway hyper‐responsiveness (AHR) compared to those that received the single treatment at the highest MCh dose (*P = *0.003), and the effect was similar to that of mice that received Dex treatment. Additionally, Clo/Mon significantly suppressed the increased total cell (*P = *0.005) and eosinophil counts (*P < *0.001) (Figure 1)

### 3.2 Clo/Mon decreases the levels of Th2 cytokines and platelet and eosinophil activation markers

**Figure 2:**
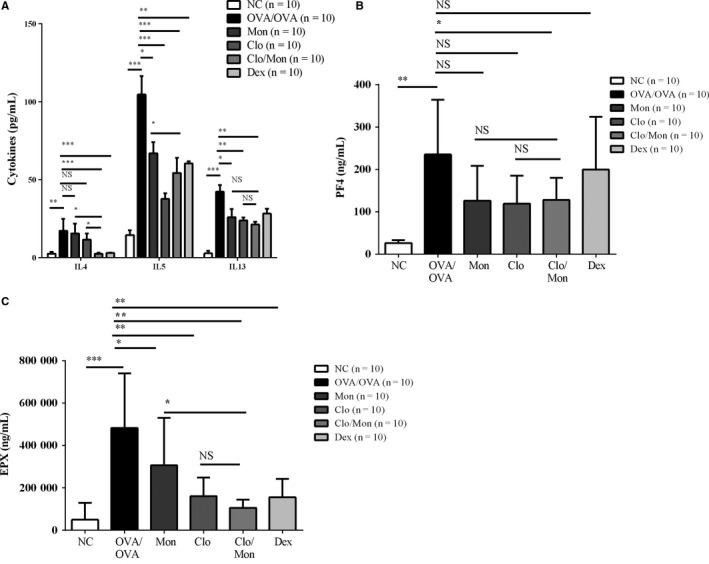
Effects of clopidogrel and montelukast (Clo/Mon) on Th2 cytokines and the activation of platelets and eosinophils. Bronchoalveolar lavage (BAL) fluid was harvested and stored at −70°C until further analysis. The BAL fluid levels of (A) interleukin (IL)‐4, IL‐5, IL‐13; (B) platelet factor 4 (PF4); and (C) EPX were measured by enzyme‐linked immunosorbent assay. Data are means ± SD. N = 10 mice per group. *P *values were analysed by one‐way ANOVA with Tukey's post hoc test, except the IL‐4 and PF4 levels were analysed by the Mann‐Whitney *U*‐test. *, **, and *** indicate *P < *0.05, 0.01 and 0.001, respectively, in the comparisons between the indicated groups

Compared to the OVA/OVA challenge, Clo/Mon suppressed the increase in IL‐4 more effectively than the single treatment and was similar to Dex (*P < *0.001; *P = *0.001) (Figure 2A). Clo/Mon reduced IL‐5 and IL‐13 levels significantly (*P < *0.001), but the effect was not different from that of a single agent therapy. Interestingly, as demonstrated by decreases in PF4 and EPX (*P = *0.016, *P = *0.001) (Figure 2B‐C), Clo/Mon significantly suppressed the activation status of platelets and eosinophils respectively.

### 3.3 Clo/Mon reduces the number of inflammatory cells and the mucus production in lung tissues

**Figure 3:**
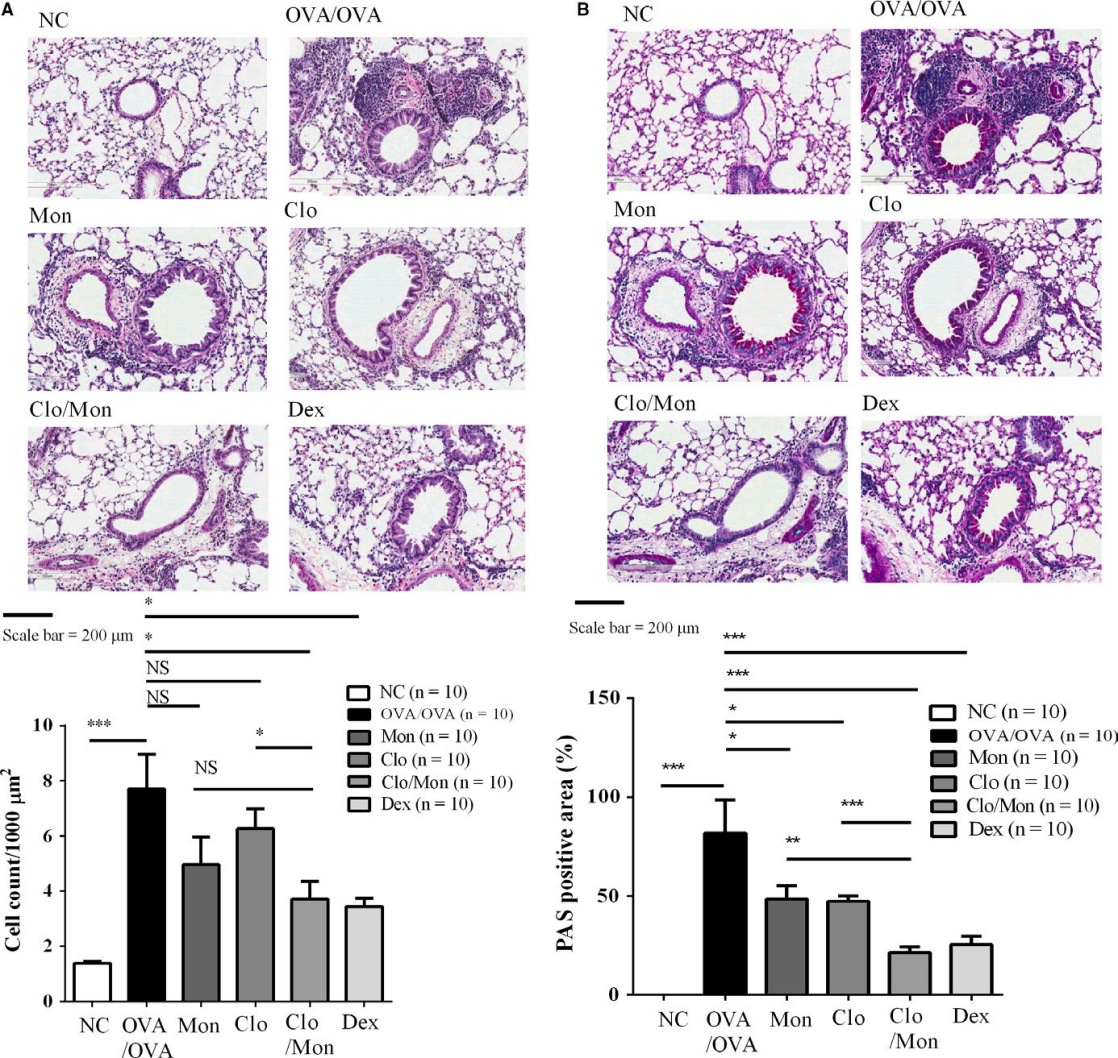
Effects of clopidogrel and montelukast (Clo/Mon) in lung tissue. A, Lung tissues were stained with haematoxylin and eosin (H&E) for inflammation and (B) with Periodic acid‐Schiff (PAS) for mucus‐containing cells. *P* values were analysed by one‐way ANOVA with Tukey's post‐hoc test. *, **, and *** indicate *P < *0.05, 0.01 and 0.001, respectively, in the comparisons between the indicated groups; NS, not significant

Clopidogrel and montelukast attenuated the number of inflammatory cells in lung tissues (*P < *0.05) (Figure 3A). Additionally, in the asthmatic mice, Clo/Mon improved mucus secretion more effectively than the single treatment (*P < *0.05) (Figure 3B).

### 3.4 Suppressive effect of Clo/Mon on PEA formation is superior to that of Dex

**Figure 4:**
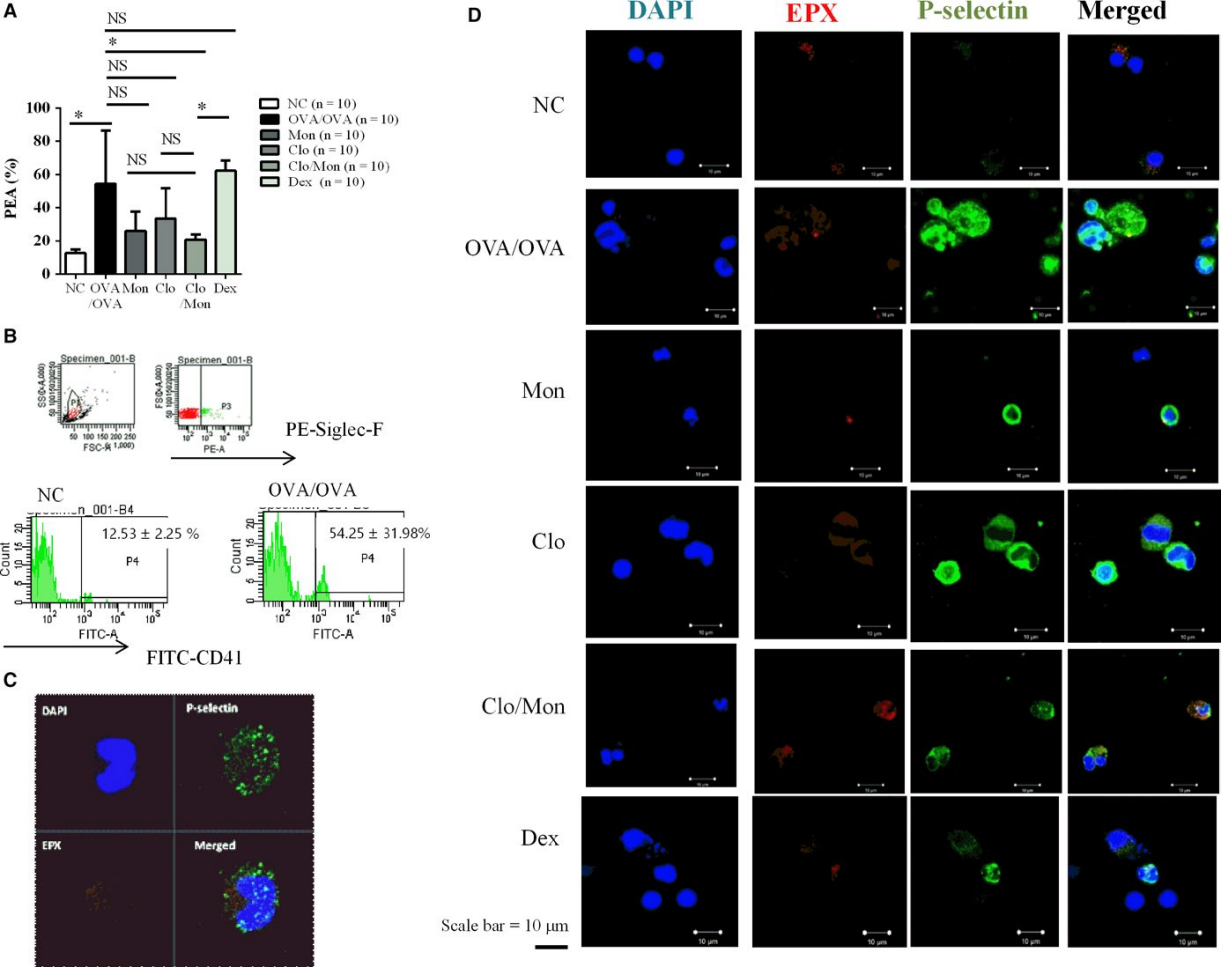
Up‐regulation of platelet‐eosinophil aggregation (PEA) in asthma. A, The percentage of PEA in mouse whole blood. Platelet‐eosinophil aggregation was defined as Siglec‐F^+^/CD41^+^ cells based on flow cytometry. Data are means ± SD *P *values were analysed by one‐way ANOVA with Tukey's post hoc test. N = 5 for each group. B, Representative image of the flow cytometric data. From the leucocytes gate (P1), eosinophils were selected based on Siglec‐F+ cells (P3). Gate P4 contains CD41+ eosinophils (Siglec‐F+/CD41+ cells). C, In whole blood and (D) bronchoalveolar lavage fluid, the activated platelets (P‐selectin) bound to the eosinophil surfaces were identified by P‐selectin and EPX. Representative images from at least three independent experiments are shown

Platelet‐eosinophil aggregation in mouse whole blood was visualized based on the cells containing P‐selectin and EPX (Figure 4). The increased level of PEA in asthma was abrogated by Clo/Mon in mouse whole blood (*P = *0.014) and BAL fluid. Dex did not affect PEA formation. The suppressive effect of Clo/Mon was stronger than that of Dex (*P = *0.023).

### 3.5 ADP and LTE_4_ induce PEA formation, which was suppressed by Clo/Mon

**Figure 5:**
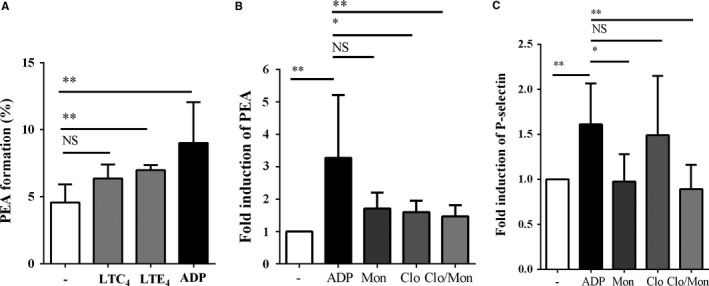
Effects of clopidogrel and montelukast (Clo/Mon) on the ligands of platelet‐eosinophil aggregations (PEA) in vitro. A, The percentages of Siglec‐F+/CD41+ cells were calculated. Data are means ± SD. B, Clopidogrel and montelukast significantly reduced PEA formation and (C) P‐selectin expression on platelets. Data are presented as folds of induction (means ± SD) compared to untreated group, from three independent experiments with duplicates. *P *values were analysed with the Wilcoxon signed‐rank test. * and ** indicate *P < *0.05 and 0.01, respectively, in the comparison between the indicated groups; NS, not significant

Leukotriene E4 and ADP induced significant formation of PEA in the in vitro assay (Figure 5A) (*P = *0.003, *P = *0.004 respectively). Adenosine diphosphate stimulated a fold induction in P*‐*selectin (*P* = 0.008), but LTE_4_ did not trigger significantly P‐selectin expression (data not shown). LTC_4_ tended to induce PEA. Clopidogrel and montelukast significantly suppressed the ADP‐induced PEA aggregation (*P = *0.008) and the expression of P‐selectin (*P = *0.003) (Figure 5B,C).

### 3.6 Clo/Mon diminishes BAL fluid and serum levels of LTE_4_

**Figure 6:**
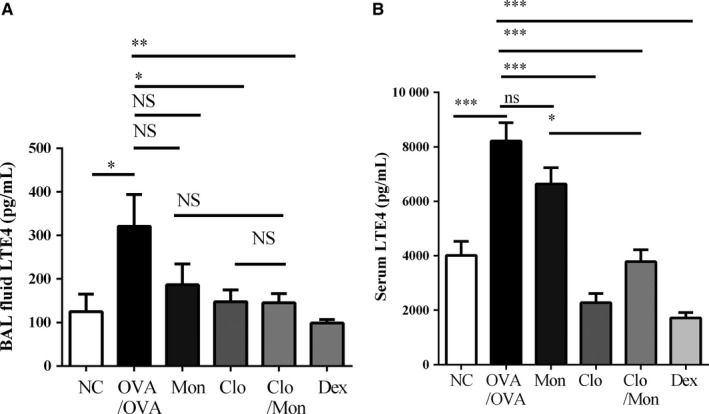
Additional effects of clopidogrel and montelukast (Clo/Mon) on reducing leukotriene E4 (LTE_4_)_._ The levels of LTE_4_ in (A) bronchoalveolar lavage fluid (BALF) and (B) serum were measured by ELISA. *P* values were analysed by one‐way ANOVA with Tukey's post hoc test. Data are means ± SEM; n = 10 per group for BALF and n = 5 per group for serum. * and ** indicate *P < *0.05 and 0.01, respectively, in comparisons between the two groups; NS, not significant

Leukotriene E4 levels increased in BAL fluid (*P = *0.025) and serum (*P < *0.001) in OVA/OVA‐challenged mice compared to normal control mice. Clopidogrel and montelukast and Dex significantly suppressed the elevated LTE_4_ levels in BAL fluid (Clo/Mon, *P = *0.048, Dex, *P = *0.005) and serum (*P < *0.001 for each). Clo/Mon was more effective at increasing serum LTE_4_ than a single treatment of Mon (*P = *0.007), but it was not superior to a single treatment with Clo (Figure 6).

### 3.7 Correlation of PEA and eosinophil count with inflammatory cytokines/chemokines and LTE_4_

**Figure 7:**
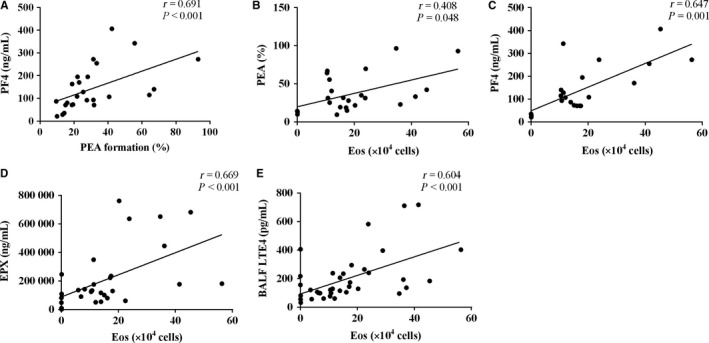
Correlations between platelet‐eosinophil aggregations (PEA) and eosinophil counts with inflammatory mediators and leukotriene E4 (LTE_4_). Platelet‐eosinophil aggregations correlated with (A) bronchoalveolar lavage fluid (BALF) level of platelet factor 4 (PF4) and (B) eosinophil count in BALF. The eosinophil count also correlated with BALF levels of (C) PF4, (D) eosinophil peroxidase (EPX), and (E) LTE_4_. Analyses were performed by Spearman's rank correlation coefficient

As in Figure 7, percentage of PEA in mouse whole blood correlated positively with eosinophil count (*r* = 0.408, *P* = 0.048) and BALF level of PF4 (*r* = 0.691, *P* < 0.001). Eosinophil count correlated with both PF4 (*r* = 0.647, *P* = 0.001) and eosinophilic cationic protein (*r* = 0.669, *P* < 0.001). Despite that BALF levels of LTE_4_ correlated positively with eosinophil count (*r* = 0.604, *P < *0.001), there were no correlations between serum LTE_4 _with cytokines/chemokines or eosinophil count.

## 4 DISCUSSION

In the current study, we demonstrated the synergistic effect of Clo and Mon on the reduction of airway inflammation. The combination of Clo/Mon was more effective than therapy with either Mon or Clo. Clopidogrel and montelukast impaired PEA formation, platelet activation mediated by ADP and platelet‐dependent eosinophil degranulation.

Clopidogrel and montelukast showed a similar effect of attenuating the AHR and IL‐4, IL‐5 and IL‐13 levels compared to Dex, but Dex was able to suppress the eosinophil count and the changes in histological structure. These results are relevant because (a) lower concentrations of drugs were used; and (b) the secondary challenge in the eosinophilic asthma mouse model. Based on the CysLTR1 to P2Y12R ratio in our previous study,^25^ we combined Mon and Clo (Clo/Mon) at a ratio of 1:1. We used considerably lower doses of Mon and Clo (10 mg/kg for each drug) compared to previous experimental studies.^18^, ^26^, ^27^ The secondary, eosinophilic asthma mouse model manifested higher eosinophilic infiltration than the model that we used previously to clearly detect the therapeutic effects of Clo/Mon. The Clo/Mon treatment suppressed the lymphocyte count in BAL fluid, which explained the decreased levels of Th2 cytokines.

In agreement with the hypothesis, Clo/Mon synergistically inhibited not only PEA formation but also activation of platelets (PF4) and eosinophils (EPX). The symbiotic association between platelets and eosinophils was reinforced in our study. Platelets and eosinophils interact through direct contact via surface ligands (eg, P‐selectin) or soluble mediators, leading to cellular activation. Platelets express both high‐ and low‐affinity IgE receptors, which may be functional in asthma.^1^, ^2^, ^28^ Numerous studies have demonstrated increased PEA after an allergen challenge in asthmatics.^29^, ^30^ Platelet and eosinophil activities correlate positively in asthmatic patients and negatively with asthma‐related quality of life.^30^ In the current study, the increase in PEA formation was impaired by Clo/Mon, possibly resulting in reduced eosinophil recruitment to lung tissues. Moreover, the adherent platelets from the PEA were more activated, leading to the release of PF4, which further triggers the activation and accumulation of eosinophils.^7^, ^31^ Indeed, the percentage of PEA correlated positively with PF4 level and eosinophil count. We found increased levels of PF4 (CXCL4) and EPX released from the α‐granules of activated platelets, which is a known potent eosinophil chemoattractant and an augmentative agent for eosinophil adhesion.^2^, ^32^ In our study, the increased PF4 in asthmatic mice enhanced the eosinophilia, which might amplify EPX production although we could not find any direct relationship between EPX and PF4.Notably, PF4 and EPX contribute directly to the induction of airway hyperactivity in animal models.^33^, ^34^ Thus, we speculate that the inhibitory effects of Clo/Mon may rely on interactions between platelets and eosinophils.

Next, we investigated the mechanism by which Clo/Mon exerted their effects. Several stimulators of platelet activation have been reported, including ADP and LTC_4_.^35^, ^36^ Adenosine diphosphate was the most potent agonist to induce PEA formation and P‐selectin overexpression, followed by LTE_4_. We could not demonstrate the effects of LTC_4_ on platelet activation, which were demonstrated previously,^35^ possibly due to the prolonged activation of platelets during the induction period in our asthma mouse model. Clopidogrel and montelukast suppressed ADP‐induced PEA formation and P‐selectin expression. Adenosine diphosphate is known to stimulate platelets thorough a phosphoinositide 3‐kinase (PI3K)/Akt‐dependent pathway.^37^, ^38^ Both P2Y12 and CysLTR1 are involved in the PI3K/Akt pathway,^36^, ^40^ which strongly suggests the synergistic effects of the two respective antagonists Clo/Mon. P2Y12R antagonists inhibit eosinophil degranulation.^17^ Therefore, an addition of P2Y12R supports the effect of Mon in suppressing eosinophil protease activity and infiltration.^41^ It is intriguing that LTE_4_ also induced PEA formation independent of the P‐selectin ligand. We determined that LTE_4_ levels increased significantly in asthmatic mice, which is inline with previously reported results.^42^ Eosinophil count correlated positively with BALF LTE_4, _which reinforce that eosinophils are the major source of LTE_4. _The increased level of LTE_4_ could also be a consequence of PEA formation because LTC_4_ synthase from platelets amplifies CysLT production by eosinophils,^7^ subsequently rendering PEA by another mechanism, such as through a P2Y12R‐ or CysLTR1‐dependent pathway. Suppressing the increase in LTE_4_ and PEA by Clo/Mon is more relevant in aspirin‐exacerbated respiratory disease, which is a severe clinical phenotype of asthma characterized by LT overproduction and eosinophilia.

In conclusion, Clo operated synergistically with Mon to diminish the platelet‐eosinophil interaction and platelet‐dependent eosinophil recruitment and degranulation, thereby attenuating airway inflammation and LTE_4_ production from eosinophils in the asthma model. This process was mediated by ADP and supported by increased LTE_4 _production. The combination of the two antagonists may be potential in asthma treatment, particularly for more severe eosinophilic asthma.

## CONFLICT OF INTEREST

The authors confirm that there is no conflict of interest.
