# Supplementary figures and images for: The synergistic effects of clopidogrel with montelukast may be beneficial for asthma treatment
Source: J Cell Mol Med. 2019 Mar 23;23(5):3441–50. doi: 10.1111/jcmm.14239 (PMC6484307; doi:10.1111/jcmm.14239)

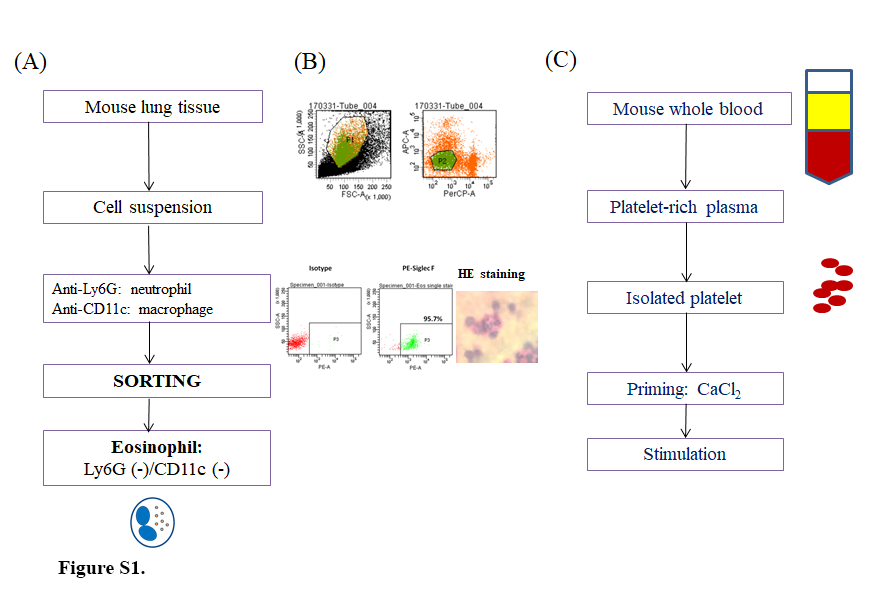

Supplement: Supplementary file 1 [file JCMM-23-3441-s001.tif]
