# Supplementary material for: The synergistic effects of clopidogrel with montelukast may be beneficial for asthma treatment
Source: J Cell Mol Med. 2019 Mar 23;23(5):3441–50. doi: 10.1111/jcmm.14239 (PMC6484307; doi:10.1111/jcmm.14239)
Supplement: Supplementary file 2 [file JCMM-23-3441-s002.doc]

**The Synergistic Effects of Clopidogrel with Montelukast may be Beneficial for Asthma Treatment**

(**Running title:** Additional effects of clopidogrel and montelukast in asthma)

Hoang Kim Tu, Trinh, MD^1^; Thuy Van Thao, Nguyen; MD, MS^2^; Youngwoo Choi, PhD^1^; Hae-Sim, Park, MD, PhD^1, 3^; Yoo Seob, Shin, MD, PhD^1^.

1: Department of Allergy and Clinical Immunology, Ajou University School of Medicine, Suwon, South Korea.

2: Department of Pediatrics, University of Medicine and Pharmacy at Ho Chi Minh city, Vietnam.

3: Department of Biomedical Science, Ajou University School of Medicine, Suwon, South Korea.

# **Corresponding authors:**

Yoo Seob Shin, MD, PhD, Department of Allergy & Clinical Immunology, Ajou University School of Medicine, Worldcup-ro 164, Yeoungtong-gu, Suwon-si, Korea, 443-380

Tel: +82-31-219-5150

Fax: +82-31-219-5154

E-mail: [drsys93@naver.com](mailto:drsys93@naver.com)

**SUPPLEMENTARY FIGURES**

**Fig. S1. Platelet and eosinophil enrichment scheme.** (A) Eosinophils were isolated from homogenized mouse lung tissues. The cell suspension was labeled with PerCP-conjugated anti-Ly6G antibody and APC-conjugated anti-CD11c antibody. The cells were sorted by flow cytometry. A leukocyte gate was set based on the FSC/SSC characteristics. The eosinophils were defined as Ly-6G-/CD11c-leukocytes. (B) Representative images of the flow cytometric data and hematoxylin-eosin-stained samples are shown. (C) Platelets were isolated from mouse whole blood by centrifugation. The PRP was centrifuged one more time to isolate the platelets.
